# Correlation between imaging features and rEm18 antibodies in alveolar echinococcosis: results from a multicenter study in France

**DOI:** 10.1051/parasite/2024076

**Published:** 2025-02-07

**Authors:** Gabriel Simon, Frédéric Grenouillet, Carine Richou, Eric Delabrousse, Oleg Blagoskonov, Anne Minello, Gerard Thiefin, Emilia Frentiu, Martine Wallon, Solange Bresson-Hadni, Paul Calame

**Affiliations:** 1 Department of Radiology, University Hospital of Besançon 25030 Besançon France; 2 Research Unit “Synergie”, University of Franche-Comté 25030 Besançon France; 3 UMR CNRS “Chrono-environnement”, University of Franche-Comté 25030 Besançon France; 4 Laboratory of fungi and parasite serology, University Hospital of Besançon 25030 Besançon France; 5 National Reference Center for Echinococcoses, Department of Parasitology-Mycology, University Hospital of Besançon 25030 Besançon France; 6 Department of Hepatology, University Hospital of Besançon 25030 Besançon France; 7 Department of Nuclear Medicine, University Hospital of Besançon 25030 Besançon France; 8 Department of Hepatology, University Hospital of Dijon 21000 Dijon France; 9 Department of Hepato-Gastroenterology and Digestive Oncology, University Hospital of Reims 51100 Reims France; 10 Department of Infectious Diseases, University Hospital of Caen 14000 Caen France; 11 Department of Parasitology and Medical Mycology, University Hospital of Lyon 69002 Lyon France; 12 EchinoVISTA Group: Frédéric Dalle, Sandrine Gohier, Pascal Chavanet (University Hospital of Dijon); Jérome Dumortier (University hospital of Lyon); Valérie Laurent, Lorraine Letranchant, Jérome Watelet, Marie Machouart, Anne Debourgogne (University Hospital of Nancy); Christine Hoeffel, Cathy Chemla, Thomas Feron, Daniele Sommacale, Alexandra Heurge (University Hospital of Reims); Yves Hansmann, Nicolas Lefebvre, Ahmed Abou-Bacar, Ermano Candolfi (University Hospital of Strasbourg); Delphine Weil-Verhoeven, Claire Vanlemmens, Bruno Heyd, Sophie Felix, Damien Montange, Florence Grenouillet (University Hospital of Besançon)

**Keywords:** Alveolar echinococcosis, Positron emission tomography with computed tomography, Albendazole, rEm18-ELISA, Magnetic resonance imaging

## Abstract

*Background*: To correlate imaging features of alveolar echinococcosis (AE) with the antibodies to recombinant Em18 (rEm18AB) at diagnosis and evaluate initial imaging features associated with serologic response, this retrospective study used data from the prospective multicenter EchinoVISTA study (NCT02876146). Imaging and serology were performed at diagnosis and during follow-up. Univariate and multivariate analyses were used to evaluate imaging features associated with the rEm18AB index. Follow-up analyses evaluated the imaging features associated with serologic response (defined as a 50% reduction in the baseline value within 2 years) in non-operated patients treated with albendazole alone. *Results*: From June 2012 to July 2016, 45 patients were included, with 8/45 (18%) having an rEm18AB index < 1. Maximum lesion size (76 mm [IQR = 57–93] *vs.* 36 mm [IQR = 26–51], *p* = 0.006), microcyst percentage (70% [IQR = 3–8] *vs*. 20% [IQR = 0.5–3.5], *p* = 0.004), and maximum standardized uptake value (SUV) on fluorodeoxyglucose positron emission tomography (5.1 [IQR = 4.4–6.2] *vs*. 2.6 [IQR = 2.4–3.9], *p* = 0.001) were associated with an rEm18AB index > 1. In patients treated with albendazole, serologic responders at 2 years had smaller lesions (5.3 [IQR = 3.8–72] *vs*. 3.5 [IQR = 2.7–3.7], *p* = 0.010) with less pedicle involvement, and lower initial rEm18AB index (2.98 ± 1.63 *vs*. 7.81 ± 3.95, *p* = 0.011). *Conclusion*: Maximum lesion size, percentage of microcysts within the lesion, and maximum lesion SUV are significant imaging features of AE correlated with the rEm18AB index. Serologic response at 2 years occurs primarily in patients with small lesions and a low rEm18AB index.

## Abstract (fr)

*Contexte* : Pour corréler les caractéristiques d’imagerie de l’échinococcose alvéolaire avec les anticorps anti-Em18 recombinant (rEm18AB) au moment du diagnostic et évaluer les caractéristiques d’imagerie initiales associées à la réponse sérologique, cette étude rétrospective a utilisé les données de l’étude prospective multicentrique EchinoVISTA (NCT02876146). L’imagerie et la sérologie ont été réalisées au moment du diagnostic et pendant le suivi. Des analyses univariées et multivariées ont été utilisées pour évaluer les caractéristiques d’imagerie associées à l’indice rEm18AB. Les analyses de suivi ont évalué les caractéristiques d’imagerie associées à la réponse sérologique (définie comme une réduction de 50 % de la valeur initiale dans les 2 ans) chez les patients non opérés traités par albendazole seul. *Résultats* : De juin 2012 à juillet 2016, 45 patients ont été inclus, dont 8/45 (18 %) avaient un index rEm18AB < 1. La taille maximale des lésions (76 mm [IQR = 57–93] contre 36 mm [IQR = 26–51], *p* = 0,006), le pourcentage de microkystes (70 % [IQR = 3–8] contre 20 % [IQR = 0,5–3,5], *p* = 0,004) et la valeur maximale de captation standardisée (SUV) sur la tomographie par émission de positons au fluorodésoxyglucose (5,1 [IQR = 4,4–6,2] contre 2,6 [IQR = 2,4–3,9], *p* = 0,001) étaient associés à un index rEm18AB > 1. Chez les patients traités par albendazole, les répondeurs sérologiques à 2 ans présentaient des lésions plus petites (5,3 [IQR = 3,8–72] contre 3,5 [IQR = 2,7–3,7], *p* = 0,010) avec une atteinte pédiculaire moindre et un indice rEm18AB initial plus faible (2,98 ± 1,63 contre 7,81 ± 3,95, *p* = 0,011). *Conclusion* : La taille maximale de la lésion, le pourcentage de microkystes au sein de la lésion et le SUV maximal de la lésion sont des caractéristiques d’imagerie significatives de l’échinococcose alvéolaire corrélées à l’indice rEm18AB. La réponse sérologique à 2 ans survient principalement chez les patients présentant de petites lésions et un faible indice rEm18AB.

## Introduction

Alveolar echinococcosis (AE) is a rare zoonotic disease caused by infection with the larval stage (metacestode) of the parasite *Echinococcus (E.) multilocularis* [27, 26]. AE is endemic in the northern hemisphere, mainly in China and central Asia [1], central-Western Europe [22], and the Northern part of North America [26]. Diagnosis of AE relies on medical imaging combined with immunodiagnostic testing. According to international recommendations, treatment of AE includes curative surgical resection of the lesions, whenever possible, with associated albendazole (ABZ) treatment for 2 years, or ABZ alone until parasite viability markers have disappeared [27].

Immunodiagnosis involves primary screening, based mainly on enzyme-linked immunosorbent assays (ELISA), followed by confirmatory tests for specificity, such as immunoblotting [12]. Common serologic tests include *E. granulosus*-ELISA, and *E. multilocularis*-ELISA using *E. multilocularis* crude antigen, Em2-antigen, or recombinant (r) Em18-antigen [2, 4, 14, 16]. ELISA using the recombinant antigen rEm18 offers high sensitivity (96.7%) and specificity (91.5%) for AE immunodiagnosis [15, 17, 19, 24, 25]. Quantitative assessment and follow-up of the immune response to specific antigens considered to be markers of *E. multilocularis* viability, such as rEm18 antigens, have been recommended to assess treatment efficacy after curative surgery or long-term ABZ therapy [3]. Since 2018, a commercially available kit using rEm18 antigen to assay anti-Em18 antibodies (AB) (rEm18-ELISA^®^ kit, Bordier Affinity Products) has facilitated comparisons of results across studies; however, only one study has evaluated this reagent to date [13].

In AE, parasitic viability is an important parameter for planning the therapeutic strategy. Currently, ^18F^fluorodeoxyglucose positron emission tomography – computed tomography (^18F^FDG PET-CT) is the most frequently used modality to approach *E. multilocularis* viability, by measuring ^18F^FDG uptake by the cells of the periparasitic granuloma [8, 23]. Significantly increased uptake of the ^18F^FDG by AE lesions is now considered to be a marker of the “metabolic activity” of the lesions, and a surrogate marker of parasite viability [1, 8, 14]. Other cross-sectional imaging techniques, more easily available in all settings, are used to assess the diagnosis of AE and to determine the extension of the lesions [5, 7]. Computed tomography (CT) is used to analyze the presence of calcifications and the extension of AE lesions in the liver and to other organs. Magnetic resonance imaging (MRI) shows the pathognomonic microcysts in T2-weighted imaging [1, 5], thus confirming the diagnosis of AE.

Imaging-based classifications have been proposed to help clinicians in their diagnosis and therapeutic decisions, such as the World Health Organization’s Parasite, Neighboring Organs, Metastasis (PNM) index and staging [18]. However, this type of classification does not include any markers of metabolic activity of the disease, and/or of parasite viability. Kodama [20] or Kodama XUUB [5] classifications are based on MRI. The presence of microcysts in the lesions, which underlies these classifications, has been shown to significantly correlate with ^18F^FDG uptake by the lesions and has been proposed as a surrogate marker of *E. multilocularis* viability [1]. International recommendations have proposed the combination of imaging and anti-rEm18AB measurement as the best approach to assess parasite viability and guide the clinician’s decision to discontinue treatment, after surgery or long-term ABZ treatment [11]. However, correlations between the various imaging techniques and levels of rEm18AB, assessed by the commercially available rEm18^®^ kit, have rarely been explored [13].

The main aim of this study was to retrospectively evaluate the correlation between various imaging features (MRI, CT, and PET-CT) and the level of specific anti-rEm18AB, expressed by the serologic “rEm18AB index”, at the time of AE diagnosis among patients in a prospective cohort. The secondary aim was to evaluate initial imaging features associated with serologic response.

## Materials and methods

### Ethics

This study was conducted in accordance with the Declaration of Helsinki. The study protocol was reviewed and approved by the Ethics Committee (CPP est2 #11/606). All participants received detailed information about the study’s objectives, procedures, and benefits, and provided written informed consent prior to any study-related procedures.

### Study population

Patient data were collected as part of the prospective multicenter EchinoVISTA Study (NCT02876146). It involved six University Hospitals that are part of the “FrancEchino” network [9] located in Eastern France, the most endemic area for AE in the country [24]. The EchinoVISTA study aimed to help clinicians in the management of patients with hepatic AE treated with ABZ combined or not with curative surgery. Its main objective was to determine if the combination of ^18F^FDG PET-CT and rEm18AB measurements was useful to make timely decisions regarding ABZ withdrawal, including after curative surgery. The study included the collection of clinical data, storage of sera in a biobank, and imaging follow-up over 4 years.

**Figure 1:**
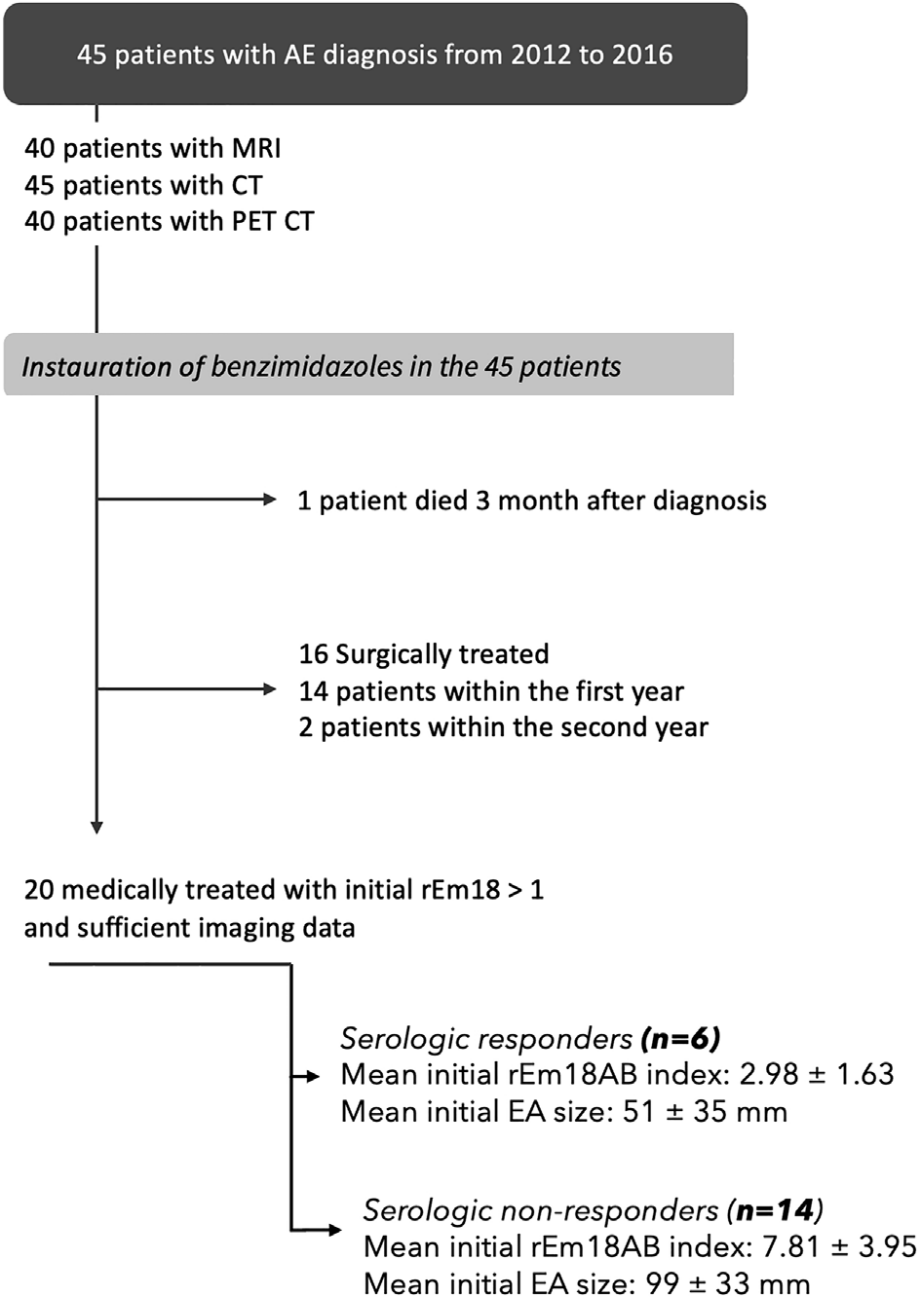
Flow chart of the study population.

Patients were included between June 2012 and July 2016. The inclusion criteria were the availability of imaging results at diagnosis, including at least one CT scan and/or liver MRI, ^18F^FDG PET-CT, AE serology, and clinical data. The initial protocol included various timings for completing the imaging and serology data, depending on the presence or absence of curative surgery. For the present study, both imaging and serology were analyzed at diagnosis and the follow-up serology was assessed at 2 years in non-operated patients treated with benzimidazoles. The exclusion criteria were absence of imaging at diagnosis, absence of serum at diagnosis or follow-up, other concomitant liver diseases, or extra-hepatic AE. Follow-up characteristics included dates of ABZ initiation, ABZ withdrawal, and surgery, and results of AE serology, imaging (CT and/or MRI) and ^18F^FDG PET-CT. Among the 54 patients included in EchinoVISTA, 9 were excluded because of incomplete imaging at diagnosis. A total of 45 patients were therefore included in the study (Figure 1). Among the 45 patients, all underwent CT at diagnosis, 40/45 (89%) liver MRI, and 41/45 (91%) ^18F^FDG PET-CT.

### Detection of rEm18AB

The commercial rEm18-ELISA^®^ kit (Bordier Affinity Products, Crissier, Switzerland) was used for all patients on the sera stored in the EchinoVISTA Biobank at the University Hospital of Besançon, France. Results were expressed as a quantitative “rEm18AB index” with two decimals and classified as positive if ≥1 or negative if <1, according to the manufacturer’s recommendations. Due to the lack of established criteria, the “serologic response” assessed by rEm18AB course was defined as a 50% reduction in the baseline value within 2 years of follow-up.

### Imaging protocols

Abdominal CT scans were performed in triple phase: unenhanced acquisition, late arterial, and portal phase. Liver MRI included T2-weighted axial sequences with and without fat suppression, T1-weighted axial sequences without and after injection of gadolinium contrast at multi-arterial and portal phases, and diffusion-weighted imaging and apparent diffusion coefficient (ADC) mapping. The ^18F^FDG PET-CT scans were acquired with an additional delayed phase (3 h post-injection), according to previous recommendations [8].

### Imaging analysis

All cross-sectional imaging studies were retrospectively reviewed by two radiologists with 10 and 4 years of experience in abdominal imaging. Image analysis was performed on Carestream PACS (Carestream Health Inc., Rochester, NY, United States). For each patient, imaging features of AE lesions were collected and categorized as follows: maximum lesion size, if single, and of the biggest AE lesion, if multiple, location of the lesions in the liver, and hepatic pedicle involvement (presence or absence). The extent of involvement of pedicle structures (hepatic artery, portal vein, and biliary tree) was graded as follows: none, segmental, sectorial, or proximal. Hepatic vein involvement was graded as none, subsegmental, segmental, or proximal. Vessel and pedicle structures were defined by the AE lesion abutment with the respective structure. CT features included the presence of microcalcifications and macrocalcifications. MRI features included the presence of microcysts, as hypersignal, and of areas in hyposignal, in the AE lesions on T2-weighted images.

The five AE types proposed by Kodama in 2003 were also recorded [20]: Type I: multiple small microcysts without a solid component; Type II: multiple small microcysts with a solid component; Type III: a solid component surrounding a large and/or irregular pseudo-cyst with multiple small microcysts; Type IV: a solid component without microcysts; and Type V: a large cyst without a solid component.

^18F^FDG PET-CT parameters included maximum lesion standardized uptake value (SUV) per region of interest (ROI) in the lesion, and healthy liver SUV per ROI in the parenchyma; lesion and liver SUV were recorded at 1 and 3 h after injection of the ^18F^FDG and their ratio was calculated for each duration of acquisition. ^18F^FDG PET-CT were retrospectively reviewed by a nuclear medicine physician (with 18 years of experience) blinded with regard to the radiologic information, and a radiologist with 10 years of experience in AE imaging.

**Figure 2:**
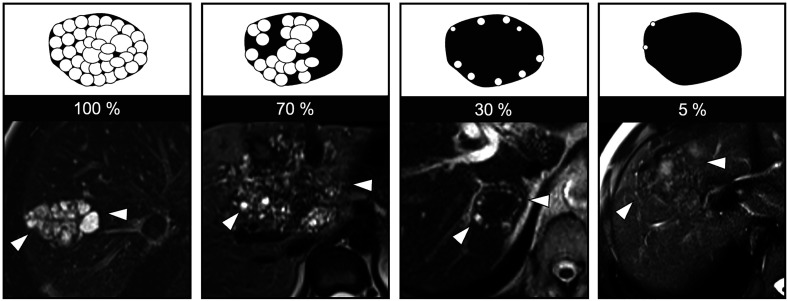
Representation of the visual assessment of microcyst percentage within an AE lesion.

The presence of microcysts on T2-weighted imaging has been highlighted as the most relevant imaging feature, with a significant correlation with ^18F^FDG uptake at PET-CT [1]. Therefore, a quantitative evaluation of the microcysts in the lesions was conducted through visual analysis. The assessment ranged from 0 to 100%, with 0% indicating no microcysts within the lesion and 100% representing a lesion entirely composed of microcysts (Figure 2).

### Statistical analysis

Continuous variables were expressed as mean ± standard deviation (SD) or median and range or interquartile range (IQR). Categorical variables were expressed as number and percentage. Comparisons between quantitative variables were performed using the Mann–Whitney *U*-test and comparison between qualitative variables by Fisher exact test or X test when appropriate. The Mann–Whitney *U-*test was used to assess the involvement of liver pedicle structures among patients with and without rEm18AB index ≥ 1 at diagnosis, and with and without serologic response at follow-up, respectively.

Correlations between the rEm18AB levels and the main imaging features were calculated using Pearson coefficient correlation, and a multivariate linear regression analysis was built to determine the variables that significantly explained the rEm18AB index.

Interrater agreements were calculated for the evaluation of microcysts percentage using the intraclass correlation coefficient, and for positive 18F-FDG PET-CT at 1 h and 3 h using Cohen’s kappa (*κ*) coefficient. A *κ* value of 0.0 indicated poor agreement; a value of 0.01–0.20, slight agreement; a value of 0.21–0.40, fair agreement; a value of 0.41–0.60, moderate agreement; a value of 0.61–0.80, good agreement; and a value of 0.81–1.00, excellent agreement.

All tests were two-sided. A *p*-value < 0.05 was considered statistically significant. All analyses were performed with R, version 3.4.4 (R Core Team 2017).

## Results

### Study population

Forty-five patients were included in the study. The mean age of the patients was 59.6 ± 15.8 years at diagnosis, with a sex ratio of 1.65 (28 men/17 women); 3/45 (7%) patients had multiple locations of AE. All the patients initially received ABZ therapy. Among them, 16 (36%) had complete (*a priori* curative) resection of the liver AE lesions within 2 years of diagnosis. One of the patients was immunosuppressed and AE was detected 1 year after bone marrow transplantation.

### Imaging features associated with rEm18 index at diagnosis

Among the 45 patients, 37 (82%) had an rEm18AB ≥ 1 at diagnosis and 8 (18%) had an rEm18AB index < 1. There were no significant differences in the mean age of patients with an index > 1 and ≤ 1 (60.9 ± 14.9 *vs*. 53.4 ± 19.2 years, respectively; *p* = 0.32). There were no differences in the gender of the patients: 25/37 (67.6%) men among patients with Em18 ≥ 1 *vs*. 3/8 (37.5%) men among patients with rEm18AB index < 1, respectively (*p* = 0.223).

Univariate analyses of the imaging features according to the rEm18AB index are summarized in Table 1. AE lesion sizes were higher in patients with rEm18AB index ≥ 1 than in patients with rEm18 index < 1 (76 [IQR = 57–93] *vs*. 36 [IQR = 26–51] mm, *p* = 0.006). In patients with rEm18AB index ≥ 1, AE lesions more likely involved hepatic artery convergence (20/37 [54%] *vs*. 2/8 [25%], *p* = 0.004), biliary convergence (25/37 [67%] *vs*. 2/8 [25%], *p* = 0.045), hepatic veins (proximal or middle vein 36/37 [97%] *vs*. 4/8 [50%], *p* = 0.001), and portal veins (proximal or middle involvement 36/37 [97%] *vs*. 6/8 [75%], *p* = 0.017).

**Table 1:** Univariate analysis of imaging features according to the rEm18AB index.

	rEm18AB index ≥ 1	rEm18AB index < 1	*p*-value
	*n* = 37	*n* = 8	
**Characteristics of AE lesions**			
Localization			0.756
Right lobe	23 (62)	5 (63)	
Left lobe	6 (16)	2 (25)	
Both right and left lobe	8 (22)	1 (13)	
Maximum size (mm)	76 [57, 93]	36 [26, 51]	**0.006**
Irregular shapes	28 (76)	5 (63)	0.746
Kodama			**0.004**
1	1 (3)	0 (0)	
2	10 (30)	1 (17)	
3	22 (67)	3 (43)	
4	0 (0)	2 (29)	
5	0 (0)	1 (14)	
Pedicle involvement	17 (46)	1 (13)	0.176
Convergence biliary involvement	25 (68)	2 (25)	**0.045**
Hepatic artery involvement	20 (54)	2 (25)	**0.004**
Portal vein involvement			**0.017**
None	1 (3)	2 (25)	
Distal	0 (0)	0 (0)	
Middle	10 (27)	4 (50)	
Proximal	26 (70)	2 (25)	
Hepatic vein involvement			**0.001**
None	1 (3)	2 (25)	
Distal	0 (0.0)	2 (25)	
Middle	19 (51)	2 (25)	
Proximal	17 (46)	2 (25)	
**CT features**			
Presence of microcalcification	36 (97)	8 (100)	1
Presence of macrocalcification	22 (59)	4 (50)	0.697
MRI features			
Presence of microcysts	33 (100)	5 (71)	**0.028**
Microcysts percentage	7 [3, 8]	2 [0.5, 3.5]	**0.004**
Hyposignal on T2 weighted imaging	30 (91)	7 (100)	0.968
**PET-CT imaging**			
Positive PET-CT (%) at 1 h	25 (76)	2 (25)	**0.014**
Positive PET-CT (%) at 3 h	29 (88)	4 (50)	**0.036**
Maximum AE lesion SUV at 1 h	5.1 [4.4, 6.2]	2.6 [2.4, 3.9]	**0.001**
Mean liver – SUV at 1 h	1.70 [1.2, 1.9]	1.7 [1.1, 1.8]	0.390
rEm18AB index	5.9 [3.5, 8.2]	0.64 [0.51, 0.79]	**<0.001**

AE = alveolar echinococcosis; CT = computed tomography; MRI = magnetic resonance imaging; PET-CT = positron emission tomography-computed tomography; SUV = standardized uptake value.

Non-normal quantitative variables are expressed as median and IQR, and compared using the Mann–Whitney *U-*test.

Normal quantitative variables are expressed as mean ± standard deviation and compared using the Student test.

Qualitative variables are expressed as numbers (percentages), and compared using χ^2^ or Fisher exact tests. Numbers in bold are significant *P* value (i.e. < 0.05).

No differences were observed for the presence of micro- or macrocalcifications (36/37 [97%] *vs*. 8/8 [100%], *p* > 0.99 and 22/37 [59%] *vs*. 4/8 [50%], *p* = 0.697, respectively). The intraclass correlation coefficient between Reader 1 and Reader 2 for microcyst percentage was 0.84 (95% CI: 0.74–0.91). In patients with rEm18AB index ≥ 1, using MRI, microcysts were significantly more frequently observed in AE lesions and the percentage of area occupied by the microcysts in the lesions was higher (33/33 [100%] *vs*. 5/7 [71%], *p* = 0.028 and 70% [IQR = 30–80] *vs*. 20% [5%–35%] respectively, *p* = 0.004).

No differences were observed for the presence of areas in hyposignal on T2-weighted sequences (30/33 [91%] *vs*. 7/7 [100%], *p* = 0.968). AE lesions were significantly more often classified as “active” according to Kodama classification (type 1, 2 or 3) in the patients with rEm18AB index ≥ 1 (type 1: 1/33 [3%] *vs*. 0/7 [0%], type 2: 10/33 [27%] *vs*. 1/7 [17%], type 3: 22/33 [67%] *vs*. 3/7 [43%]). They were more frequently classified ‘inactive’ (types 4 or 5) in patients with rEm18AB index < 1 (type 4: 0/33 [0.0%] *vs*. 2/7 [29%], type 5: 0/37 [0.0%] *vs*. 1/7 [14%]) (*p* = 0.004).

Kappa coefficient correlation for the decision on positive “metabolic activity” from the ^18F^FDG PET-CT assessment was 0.70 at 1 h (95% CI: 0.46–0.84) and 0.82 at 3 h (95% CI: 0.61–0.92). In patients with an rEm18AB index ≥ 1, metabolic activity of the lesions at PET-CT was observed in 25/33 (76%) at 1 h compared to 2/8 (25%) in those with an index < 1 (*p* = 0.014), and at 3 h, positivity was found in 29/33 (88%) versus 4/8 (50%), respectively (*p* = 0.036). Maximum AE lesion SUVs at 1 h were higher in patients with an rEm18AB index ≥ 1 (5.1 [IQR = 4.4–6.2] *vs*. 2.6 [IQR = 2.4–3.9], *p* = 0.001).

**Figure 3:**
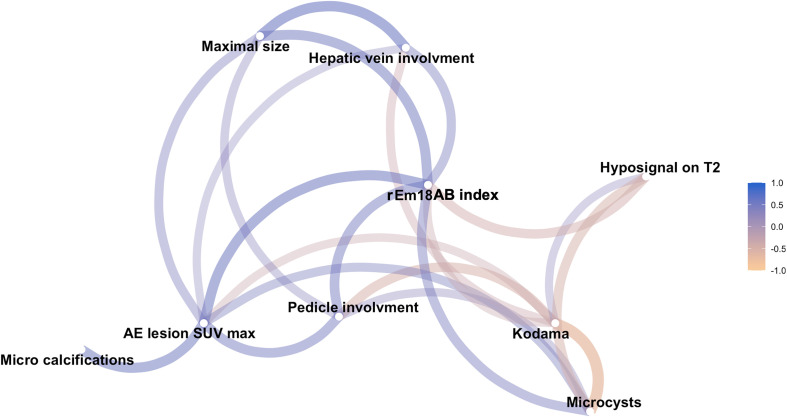
Network correlation graph showing correlations between rEm18AB and imaging features at diagnosis. Lines between nodes represent correlations, with color representing the direction of connection (*i.e.*, purple connection indicates positive correlation, yellow connection, negative correlation), with thickness showing the strength. The rEm18AB index shows a strong correlation with several imaging features, indicating its central role for parasitic viability assessment.

Correlations between the main AE imaging features and the rEm18AB index are given as a network correlation graph in Figure 3 and show moderate to good positive correlations between the rEm18AB index and maximum lesion SUV, size of the lesions, hepatic pedicle and hepatic vein involvement, and presence of microcysts, as well as moderate to good negative correlations between the rEm18AB index and Kodama types and hyposignal on T2-weighted sequences.

### Linear correlation of imaging features and rEm18AB levels

**Figure 4:**
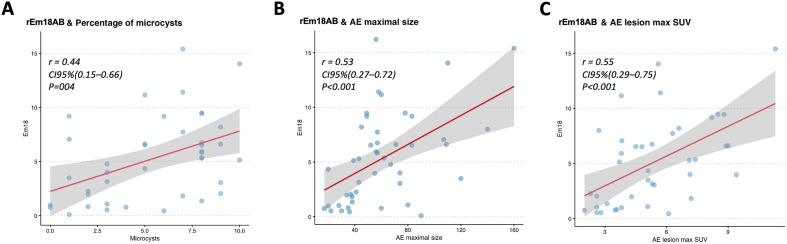
Linear correlation between the rEm18AB index and the three main imaging features. A. Correlation between rEm18AB index and percentage of microcysts. B. Correlation between rEm18AB index and maximum lesion size. C. Correlation between rEm18AB index and maximum AE lesion SUV.

Pearson coefficient correlation between the three main significant imaging features and the rEm18AB index is given in Figure 4 and highlights a moderate correlation between rEm18AB index and microcysts quantification (*r* = 0.44 [95% CI: 0.15–0.66], *p* < 0.001), maximum lesion size (*r* = 0.53 [95% CI: 0.27–0.72], *p* < 0.001) and AE maximum lesion SUV (*r* = 0.55 [95% CI: 0.29–0.75], *p* < 0.001).

Results of multivariate analysis using the three main significant imaging features, maximum lesion size, percentage of microcysts, and maximum lesion SUV are given in Table 2. The maximum lesion size and percentage of microcysts were found to be independently associated with rEm18AB levels (*p* = 0.004 and *p* = 0.016, respectively).

**Table 2:** Multivariate linear regression analysis for imaging features associated with the rEm18 index.

Variable	Beta coefficient	Standard deviation	*p*-value
Intercept	−2.59	4.064	0.073
Maximum AE lesion size	1.055	1.017	**0.004**
Percentage of microcysts	1.548	1.189	**0.016**
Maximum AE lesion SUV	1.495	1.312	0.149

AE = alveolar echinococcosis; SUV = standardized uptake value. Numbers in bold are significant *P* value (i.e. < 0.05).

### Imaging features associated with the serologic response in patients with ABZ therapy alone

**Figure 5:**
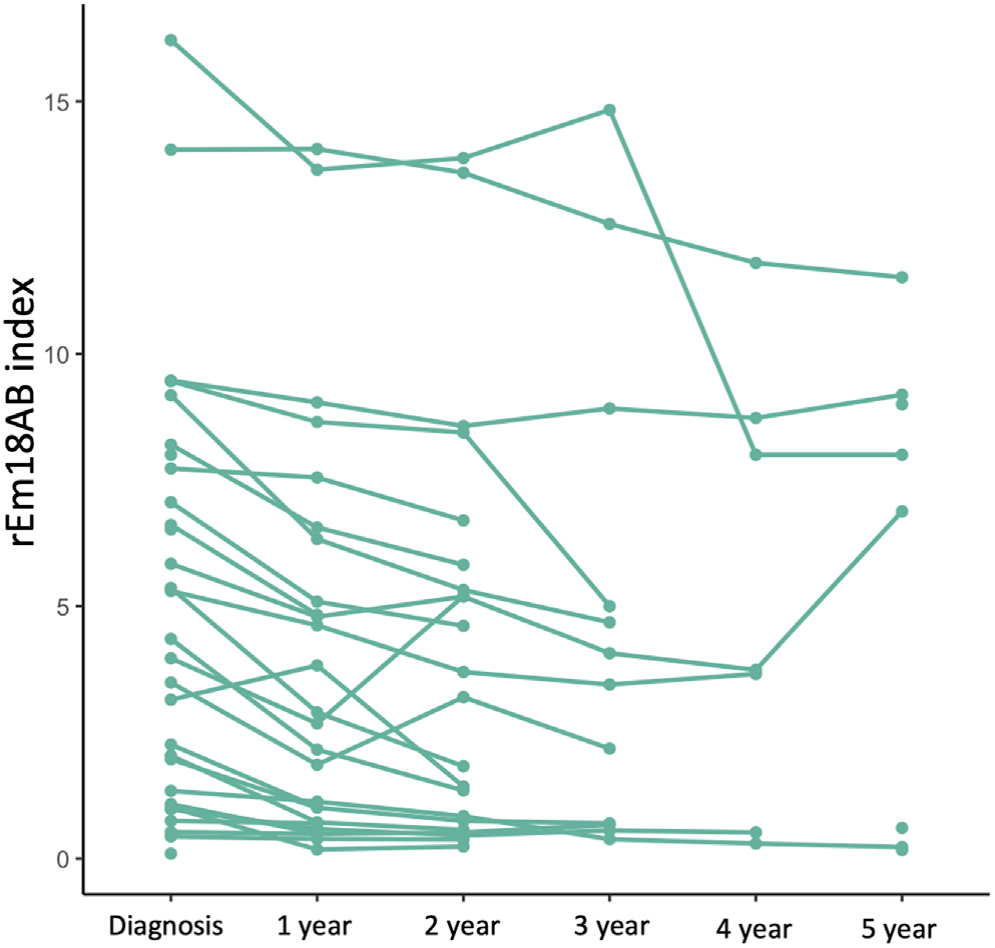
Line graph illustrating changes in the rEm18AB index in patients with ABZ therapy alone.

All 45 patients received ABZ therapy. Among them, 16 (36%) underwent curative surgical resection of the AE lesions (12 men, 4 women, 54 ± 17 years of age at diagnosis). The mean duration of serologic follow-up was 33 months in the patients treated with ABZ therapy alone. Two patients (7%) died; neither of the two deaths were related to AE. ABZ was not discontinued in any of the patients treated with antiparasitic drugs alone. Changes in rEm18AB index for all patients in this group are provided in Figure 5.

**Figure 6:**
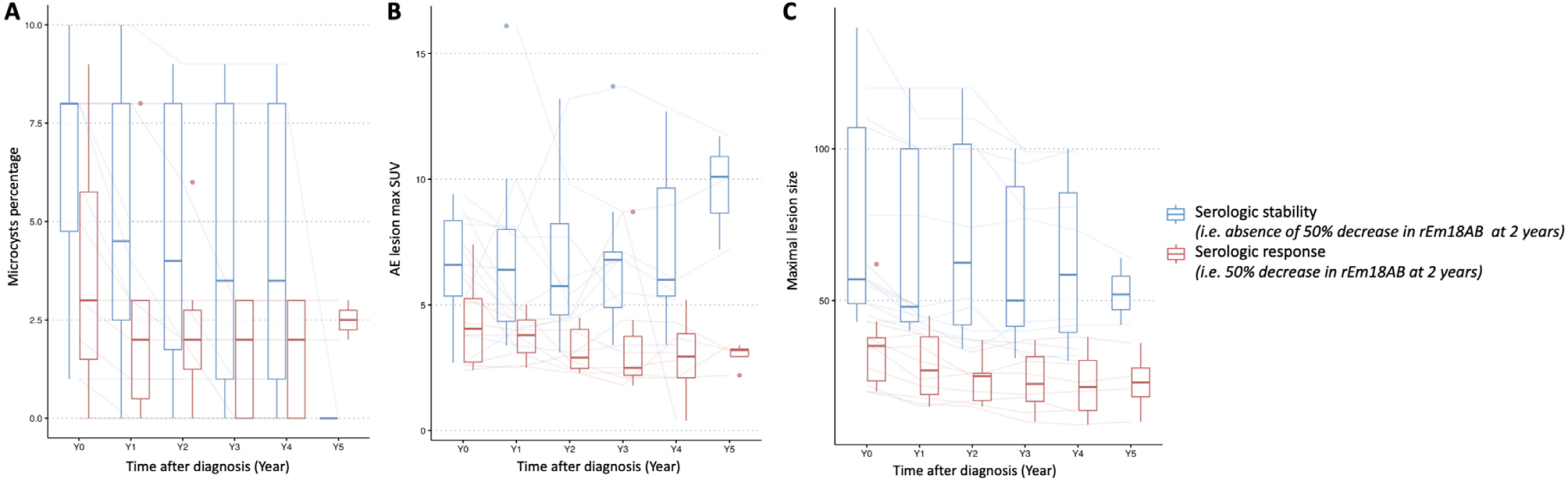
Box plot showing changes in the three main imaging features according to the serologic response. A. Change in percentage of microcysts according to the serologic response. B. Change in maximum lesion size according to the serologic response. C. Change in maximum lesion SUV according to the serologic response.

During follow-up, 20/29 (69%) patients treated with antiparasitic drugs alone were evaluated: 4 patients had insufficient imaging follow-up data and 5 had an rEm18AB index < 1 at diagnosis. Among them, 6/20 patients (33%) had a serologic response, *i.e.*, 50% reduction in rEm18AB index, at 2 years. Initial imaging features associated with serologic response to ABZ therapy are summarized in Table 3. Patients with serologic response at 2 years had significantly smaller AE lesions (mean initial maximum lesion size of 51 ± 34 *vs*. 99 ± 33 mm, *p* = 0.009), less portal vein involvement (2 [IQR = 2–2.75] *vs*. 3 [IQR = 2.25–3], *p* = 0.005), less hepatic artery involvement (1 [IQR = 0.25–2.5] *vs*. 2 [IQR = 2–2.75], *p* = 0.025), and less biliary involvement (2 [IQR = 2–2] *vs*. 3 [IQR = 3–3], *p* = 0.003) than patients without serologic response. Changes in the three main imaging features according to the serologic response of each patient are given in Figure 6.

**Table 3:** Univariate analysis of variables at diagnosis associated with serologic response at 2 years of follow-up in patients without surgery under ABZ.

	Non-responders	Responders	*p*-value
	*n* = 14	*n* = 6	
**Characteristics of AE lesions**			
Maximum size (mm)	71 ± 34	37 ± 14	**0.009**
Irregular shape (%)	11 (79)	4 (67)	0.746
Pedicle involvement (%)	10 (71)	1 (16)	**0.028**
Hepatic artery involvement (%)	14 (100)	4 (67)	0.079
Portal vein involvement (%)			**0.033**
None	0 (0)	1 (11)	
Distal	0 (0)	0 (0)	
Middle	1 (7)	2 (33)	
Proximal	13 (93)	3 (44)	
Hepatic vein involvement (%)			0.288
None	1 (7)	0 (0)	
Distal	0 (0)	0 (0)	
Middle	4 (29)	4 (66)	
Proximal	9 (64)	2 (33)	
**CT features**			
Microcalcification extension (%)	20 [13, 30]	15 [25, 27]	0.527
Macrocalcification extension (%)	0 [0, 25]	0 [0, 0]	0.692
**MRI features**			
Microcysts percentage (%)	80 [65, 80]	50 [30, 80]	0.522
T2 hyposignal percentage (%)	20 [10, 20]	20 [10, 30]	0.595
**PET-CT imaging**			
Maximum AE lesion SUV	6.3 [4.3, 8.1]	4.3 [2.4, 5.1]	0.085
Mean liver – SUV	1.30 [1.17, 1.63]	1.65 [1.30, 1.78]	0.278
rEm18AB index	7.81 ± 3.95	2.98 ± 1.63	**0.011**

AE = alveolar echinococcosis; CT = computed tomography; MRI = magnetic resonance imaging; PET-CT = positron emission tomography-computed tomography; SUV = standardized uptake value

Non-normal quantitative variables are expressed as median and IQR, and compared using the Mann–Whitney *U*-Test.

Normal quantitative variables are expressed as mean ± standard deviation and compared using the Student test.

Qualitative variables are expressed as numbers (percentages), and compared using Fisher exact tests. Numbers in bold are significant *P* value (i.e. < 0.05).

## Discussion

Based on a prospective cohort of AE patients, our retrospective analysis of the correlation between imaging and rEm18 index at diagnosis and during a 2-year follow-up showed that the three main imaging features associated with the serologic rEm18AB index were maximum lesion size, percentage of microcysts within the lesion, and maximum lesion SUV, thus confirming the association of circulating rEm18AB and specific imaging markers of parasite viability. Under ABZ therapy alone, a serologic response at 2 years was observed in one third of patients, and mainly in patients with small AE lesions with limited hepatic pedicle involvement and a low initial rEm18AB index.

Use of the rEm18AB index for the serologic monitoring of patients with AE has proven to be highly sensitive [3, 15, 25]. Our data confirm that it can now be considered an additional follow-up decision-making tool alongside ^18F^FDG PET-CT and morphologic imaging to assess the patient’s response to antiparasitic treatment and in the long-term, to discuss possible discontinuation of this treatment in non-operated patients after several years of treatment [14]. Recent studies have confirmed a significant correlation between ^18F^FDG PET-CT uptake and rEm18AB index measurements both at diagnosis and during subsequent follow-up [13], which is consistent with the results of our study. Nonetheless, the relationship between the rEm18AB index and other imaging parameters remains to be investigated comprehensively. To date, no study had connected rEm18AB serologic response with multiparametric imaging characteristics of AE lesions – such as size, hepatic pedicle involvement, presence of microcysts on T2-weighted MRI sequences, and lesion calcifications – nor had there been any assessment of changes in these imaging findings during follow-up under ABZ therapy. Interestingly, 4/33 patients (12%) with an rEm18AB index > 1 had a 3 h negative ^18F^FDG PET-CT. This underscores that parasitic viability assessment should not rely solely on ^18F^FDG PET-CT, but also integrate serology (through the rEm18AB) and morphological imaging (through microcyst visualization in T2-weighted imaging) to guide the decision for antiparasitic treatment. Additionally, considering radiation exposure in ^18F^FDG PET-CT, as well as the poor availability of this technique in most of the highly endemic areas of *E. multilocularis* infection, there is a need for non-irradiating and easily available follow-up methods, such as serology and MRI, as surrogate markers of parasite viability.

This study showed a significant correlation between the rEm18AB index and the presence and quantity of microcysts, a correlation that is underpinned by pathophysiological data. Progression of the *Echinococcus multilocularis* metacestode is marked by budding, leading to multiple microcysts that contain active metacestode [27]. This exposure of the immune system of the host to antigens specific to viable *E. multilocularis* metacestode, such as the rEm18 antigen, appears to align with the extent of the immune response. Accordingly, larger and more microcyst-rich lesions tend to be associated with greater exposure to parasitic antigens and a more pronounced immune response.

^18F^FDG PET-CT is currently considered to be the gold standard for monitoring the viability of AE by assessing the areas of host immune reactive activity, which defines the “metabolic activity” [8, 14]. In 2003, Kodama *et al.* classified AE lesions with MRI examination into five stages according to their T2 signal [20]. Since then, a relationship has been established between ^18F^FDG uptake and Kodama type 1, 2, and 3 [1]. However, a large proportion of AE lesions are classified as type 3, making Kodama classification poorly discriminating. Efforts have been made to better characterize patients according to metabolic activity assessed by ^18F^FDG PET-CT[8], and the presence of microcysts has emerged as a major feature of parasitic viability evaluation [1, 5]. Our study goes further, by showing that quantitative assessment of the percentage of microcysts within the AE lesion is correlated with the rEm18AB index and remains independently associated with the rEm18AB index after multivariable analysis.

During follow-up, features associated with serologic response were assessed, revealing that serologic response primarily occurred in patients with smaller lesions, less involvement of the hepatic hilum, and particularly lower initial rEm18AB index. These associations raise questions about the relevance of using the rEm18AB index in patients with very large lesions and a highly elevated rEm18AB index. In immunocompetent patients, our results may lead clinicians to reconsider the interpretation of the rEm18AB index at diagnosis and in the follow-up of AE patients treated with benzimidazoles, to take into account the influence of lesion size and microcyst content, as well as the initial level of rEm18AB.

Our study has several limitations, primarily the limited number of patients and the retrospective approach, despite the initial multicenter and prospective study design. The evaluation methods for the presence of microcysts and hyposignal at MRI T2-weighted sequences, and of micro- and macrocalcifications at CT were semiquantitative, which may explain the absence of an expected correlation found between the rEm18AB index and the presence of microcalcifications at CT, suggested by the findings of a previous study [6]. However, up to now, no precise method of quantification has been established to be reliable in AE. Computer-aided image analysis should be developed to improve the quantitative assessment of microcysts in all lesions of a given patient. Only one patient of our study was immunosuppressed. Such patients are more likely to have negative serology [10, 21], and the use of the rEm18AB index to monitor response to ABZ treatment is likely to be more problematic than in immunocompetent patients. Further studies are thus needed in this growing subgroup of AE patients.
